# The Canonical E2Fs Are Required for Germline Development in Arabidopsis

**DOI:** 10.3389/fpls.2018.00638

**Published:** 2018-05-15

**Authors:** Xiaozhen Yao, Huidan Yang, Yingxiu Zhu, Jingshi Xue, Tianhua Wang, Teng Song, Zhongnan Yang, Shui Wang

**Affiliations:** College of Life and Environmental Sciences, Shanghai Normal University, Shanghai, China

**Keywords:** cell cycle, E2F, megaspore mother cell, plant germline development, pollen mitosis, epigenetic control

## Abstract

A number of cell fate determinations, including cell division, cell differentiation, and programmed cell death, intensely occur during plant germline development. How these cell fate determinations are regulated remains largely unclear. The transcription factor E2F is a core cell cycle regulator. Here we show that the Arabidopsis canonical E2Fs, including E2Fa, E2Fb, and E2Fc, play a redundant role in plant germline development. The *e2fa e2fb e2fc* (*e2fabc*) triple mutant is sterile, although its vegetative development appears normal. On the one hand, the *e2fabc* microspores undergo cell death during pollen mitosis. Microspores start to die at the bicellular stage. By the tricellular stage, the majority of the *e2fabc* microspores are degenerated. On the other hand, a wild type ovule often has one megaspore mother cell (MMC), whereas the majority of *e2fabc* ovules have two to three MMCs. The subsequent female gametogenesis of *e2fabc* mutant is aborted and the vacuole is severely impaired in the embryo sac. Analysis of transmission efficiency showed that the canonical E2Fs from both male and female gametophyte are essential for plant gametogenesis. Our study reveals that the canonical E2Fs are required for plant germline development, especially the pollen mitosis and the archesporial cell (AC)-MMC transition.

## Introduction

Plant germline development, includes sporogenesis and gametogenesis, begins with the differentiation of a spore mother cell which produces haploid gametes through meiosis and mitosis (Schmidt et al., 2015). After meiosis, plant haploid spores undergo two (for sperm) or three (for egg) rounds of mitoses to form a multicellular gametophyte. These processes involve a number of cell fate determinations including cell division, cell differentiation and programmed cell death (PCD) (Drews and Yadegari, 2002; Berger and Twell, 2011; Daneva et al., 2016). The male gametogenesis takes place in anther, while the female gametogenesis occurs in ovule. An anther often produces numerous microspore mother cells. A diploid microspore mother cell is divided through meiosis into a tetrad of four haploid microspores (Preuss et al., 1994; Rhee and Somerville, 1998). Subsequently, microspore undergoes two rounds of mitoses: pollen mitosis I (PMI) and pollen mitosis II (PMII). PMI is an asymmetric mitosis producing a large vegetative cell and a smaller generative cell (Eady et al., 1995). In Arabidopsis, the generative cell undergoes a second mitosis (PMII) to give rise to two sperms, resulting in a three-celled male gametophyte (McCormick, 1993; Twell, 2011; Gomez et al., 2015). The tapetum is degenerated at the later stage of pollen development (Gomez et al., 2015). In contrast to an anther, an ovule often selects a single archesporial cell (AC) to develop into a diploid megaspore mother cell (MMC) which is divided into four haploid megaspores through meiosis (Drews and Koltunow, 2011). Three of the megaspores are degenerated. The chalazal-most megaspore undergoes mitoses and cellularization to form a seven-celled female gametophyte, composed of four types of cells: egg, synergid, central, and antipodal (Schneitz, 1999; Skinner et al., 2004; Yang et al., 2010).

In mammals, the E2F signaling pathway plays a key role in cell fate determination (Polager and Ginsberg, 2008). Plants have orthologs of all the core regulators in the E2F signaling pathway including cyclins, cyclin-dependent kinases (CDKs), CDK inhibitors (CKIs), retinoblastoma (RB), and E2Fs. Cyclins and CKIs are positive and negative regulators of CDK, respectively. RB binds E2F to inhibit its activity. CDK phosphorylates RB to release E2F. The transcription factor E2F activates genes involved in the G1-S phase transition (Polager and Ginsberg, 2009). These core regulators have been implicated in plant gametogenesis. Mutation of an A-type cyclin, CYCA1;2, leads to delayed and asynchronous cell divisions during male meiosis (Wang Y. et al., 2004). Arabidopsis has only one A-type CDK, referred to as CDKA1, which is a homolog of yeast CDC2. In the *cdka1* mutant, the female gametogenesis is not affected, whereas the male gametogenesis is significantly disrupted. As a result of the failure of PMII, a *cdka1* mature pollen produces only a single sperm cell (Nowack et al., 2006). Arabidopsis also has a single copy of the *RB* gene, referred to as *RB-RELATED 1* (*RBR1*) (Ebel et al., 2004). RBR1 is involved in both male and female gametogenesis. In the *rbr1*/*RBR1* heterozygous anther, more than 40% of pollen contain two vegetative nuclei as a result of supernumerary mitosis. The *rbr1* microspores undergo cell death after the unicellular stage (Johnston et al., 2008). Meanwhile, the *rbr1* megaspores have more than three nuclear mitotic divisions, resulting in supernumerary nuclei (up to 15) (Ebel et al., 2004; Zhao et al., 2017). In terms of the mitotic division, the phenotype of *cdka1* mutant is opposite to that of the *rbr1* mutant as the *cdka1* mutant undergoes hypoproliferation, whereas the *rbr1* mutant does hyperproliferation. Consistently, the defects of *rbr1* mutant are suppressed by the *cdka1* mutant and vice versa (Chen et al., 2009; Nowack et al., 2012). The Arabidopsis E2F family consists of eight genes. In cell proliferation, it has been found that the canonical Arabidopsis E2Fs played an antagonistic role as E2Fa and E2Fb were positive regulators, whereas E2Fc was a negative regulator (del Pozo et al., 2002, 2006; Vandepoele et al., 2002; Magyar et al., 2005). Recently, we discovered that these E2Fs played a redundant role in plant fertility as the *e2fa e2fb e2fc* triple mutant (referred to as *e2fabc*) was sterile while their single and double mutants were fertile (Wang et al., 2014; Gu et al., 2016; Wang, 2017). These data suggest that the CDK-RB-E2F core cell cycle signaling pathway plays an important role in cell fate determination during plant germline development. However, the underlying mechanism of the regulation remains unclear. In this study, we further characterized the role of the E2Fs in plant germline development to better understand the regulation of this processes.

## Materials and Methods

### Plant Material and Growth Conditions

Arabidopsis plants used in this study are in the Columbia (Col-0) background. Mutants of *e2fa* (GK-348E09), *e2fb* (SALK_103138), and *e2fc* (GK-718E12) are as described (Wang et al., 2014). The condition of growth chamber was set at 22°C under a 16-h light/8-h dark photoperiod.

### Complementation of *e2fabc* Mutant

The *E2F* genes, including *E2Fa* (AT2G36010), *E2Fb* (AT5G22220) and *E2Fc* (AT1G47870), for complementation of the *e2fabc* mutant were amplified by PCR and integrated into the SalI site of the binary vector pCAMBIA1300^1^ using the pEASY Uni-Seamless Cloning and Assembly Kit (TransGen Biotech, Beijing, China) to generate pCAMBIA1300-E2Fs. The primers used for construction of pCAMBIA1300-E2Fs are listed in Supplementary Table S1.

### Analysis of E2F Expression

The reporters were used for analyzing the expression of *E2F* genes. The construct of *pE2F:E2F-VENUS* was a translational fusion of *E2F* to *VENUS* which was driven by its native promoter (∼2 kb). *NOS* terminator and *VENUS* were amplified by PCR and consecutively inserted into the PstI-HindIII site and the SalI-PstI site of pCAMBIA1300 to generate pCAMBIA1300-VENUS. Subsequently, the genomic DNA sequence of a *E2F* gene was amplified by PCR and integrated into the SalI site of pCAMBIA1300-VENUS using the pEASY Uni-Seamless Cloning and Assembly Kit (TransGen Biotech, Beijing, China) to generate pE2F:E2F-VENUS. The primers used for construction of pE2F:E2F-VENUS are listed in Supplementary Table S1. To visualize the expression pattern of a reporter, the fluorescence was excited at 488 nm and collected with a 515∼530 nm bandpass filter using a Zeiss LSM 5 Pascal Confocal Laser Scanning Microscopy (Germany).

### Alexander Staining

The Alexander staining was performed as described (Alexander, 1969). Briefly, anthers were stained with the Alexander solution for 30 min and images were taken using an Olympus BX51 digital microscope (Japan).

### Semi-Thin Section

Floral buds were fixed and embedded in the Spurr’s epoxy resin as described (Zhang et al., 2007). The embedded materials were sectioned to 1-μm thick using an RMC Powertome XL Ultramicrotome (Tucson, AZ, United States). Semi-thin sections of anthers were stained with toluidine blue and photographed using an Olympus BX51 digital microscope (Japan).

### DAPI Staining

Pollen was stained with 4′-6-diamidino-2-phenylindole (DAPI) as described (Ross et al., 1996). Briefly, floral buds were fixed with Carnoy’s solution (ethanol: acetic acid = 3: 1) for 4 h at room temperature and washed with water. Pollen was then released from anther and stained with 0.1 μg/ml DAPI.

### Analysis of Female Gametophyte Development

The procedure used to analyze female gametophyte development was carried out as described (Christensen et al., 1997). Briefly, pistils were fixed in the fixative solution (4% glutaraldehyde and 12.5 mM cacodylic acid, pH 6.9) for 4 h. The tissues were dehydrated in a series of increasing concentrations of ethanol (10, 20, 40, 60, 80, and 95%, each for 10 min) and kept in 95% ethanol overnight. The tissues were then washed with 100% ethanol twice, each for 10 min. After dehydration, the tissues were cleared in the benzyl benzoate/benzyl alcohol (2:1) solution for 20 min. Ovules were dissected, mounted in immersion oil, and observed at the excitation wavelength 488 nm and the emission wavelength 515∼530 nm using a Carl Zeiss LSM 5 Pascal Confocal Laser Scanning Microscopy (Germany).

### Quantitative PCR (qPCR)

The qPCR was performed as described (Ma and Wang, 2016). Briefly, Arabidopsis RNA was extracted using TRIzol Reagent (Invitrogen) and measured by NanoDrop 2000 Spectrophotometer (Thermo Fisher). Five μg of RNA was treated with DNase (Ambion TURBO DNA-free Kit, Thermo Fisher). Two μg of DNase-treated RNA was used to synthesize cDNA using the TransScript Fly First-strand cDNA synthesis SuperMix (TransGen Biotech, Beijing, China). The synthesized cDNA, diluted 5 times, was used as templates. qPCR was performed using the SYBR Green Realtime PCR Master Mix (Toyobo, Japan) in Mastercycler ep realplex (Eppendorf). All genes were normalized to *TUBULIN BETA CHAIN 2* (*TUB2*). The primers used for qPCR are listed in Supplementary Table S1.

## Results

### The Arabidopsis E2Fa, E2Fb, and E2Fc Are Canonical E2F Proteins

**FIGURE 1:**
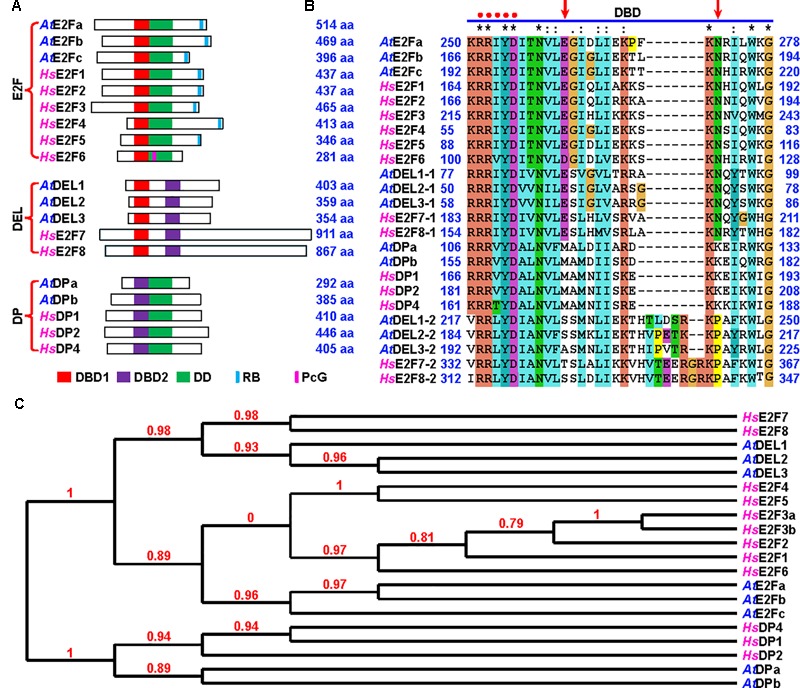
Arabidopsis E2Fa, E2Fb, and E2Fc are the canonical E2F proteins. **(A)** Schematic representation of domains of Arabidopsis and human E2F family proteins, which are classified into three categories: E2F, DEL, and DP. DBD1 and DBD2, DNA-binding domain 1 and 2; DD, dimerization domain; RB, RB-binding domain; PcG, polycomb group protein-binding domain. *At, Arabidopsis thaliana*; *Hs, Homo sapiens*. aa, amino acid residues. **(B)** Alignment of DBD of the E2F proteins using ClustalX2 (http://www.clustal.org/). Red dots indicate the core DNA recognition motif RRxYD. Arrows indicate that two amino acids, glutamate (E) and asparagine (N), in DBD1 are shared between E2F and DEL proteins. Arabidopsis DEL1, DEL2 and DEL3 and human E2F7 and E2F8 possess two DBDs: DEL1/2/3-1 and E2F7/8-1, DBD1; DEL1/2/3-2 and E2F7/8-2, DBD2. **(C)** Phylogenetic tree of plant and human E2F family proteins is constructed by the Phylogeny.fr (http://www.phylogeny.fr/). The sequences of E2F proteins in fasta format were pasted and the software was performed in “One Click” mode to generate the phylogenetic tree. The number at each branch point represents the bootstrap values.

There are three categories of E2Fs in both human and Arabidopsis based on their protein domain structures (**Figure 1A**) (Vandepoele et al., 2002; Attwooll et al., 2004). The most conserved domain in E2F proteins is the DNA-binding domain (DBD), especially the core DNA-binding motif “RRxYD” which binds to the palindromic CGCGCG sequence (**Figure 1B**) (Zheng et al., 1999). DBDs are classified into DBD1 and DBD2 (Lammens et al., 2009). We found that two amino acids, glutamate at 261 and asparagine at 272 in Arabidopsis E2Fa protein, were highly conserved in DBD1 (**Figure 1B** and Supplementary Figure S1). The first category, referred to as E2F, is the canonical E2Fs possessing a DBD1 and a dimerization domain (DD). In addition, these E2Fs have a RB-binding domain or a polycomb-group (PcG)-binding domain. The second category, referred to as DP-E2F-like 1 (DEL1), contains two DBDs: DBD1 and DBD2. The third category, referred to as dimerization partner (DP), has a DBD2 and a DD. Structural analysis demonstrated that among E2F proteins, there was a preference for heterodimers over homodimers in DNA binding (Zheng et al., 1999). Therefore, E2F and DP form a heterodimer through DD to bind DNA, whereas DELs, possessing both DBD1 and DBD2, bind DNA by themselves (**Figure 1**) (Logan et al., 2004; Lammens et al., 2009). There are eleven members of E2F family proteins in human (**Figure 1**). Based on their transcriptional properties, human E2F proteins are classified into activators, E2F1 through E2F3, and repressors, E2F4 through E2F8 (DeGregori and Johnson, 2006). Meanwhile, there are eight members of E2F family proteins in Arabidopsis (Vandepoele et al., 2002). The first category includes E2Fa, E2Fb, and E2Fc, which are the canonical E2Fs. The second category consists of DEL1, DEL2, and DEL3. The third category includes DPa and DPb (**Figure 1**). Both DBDs and three categories of E2Fs are highly conserved in plants across eudicot, monocot, lycopodiophyta, bryophyta and algae (**Figure 1B**, Supplementary Figure S1, and Supplementary Tables S2, S3).

### The Canonical E2Fs Play an Essential Role in Plant Fertility

**FIGURE 2:**
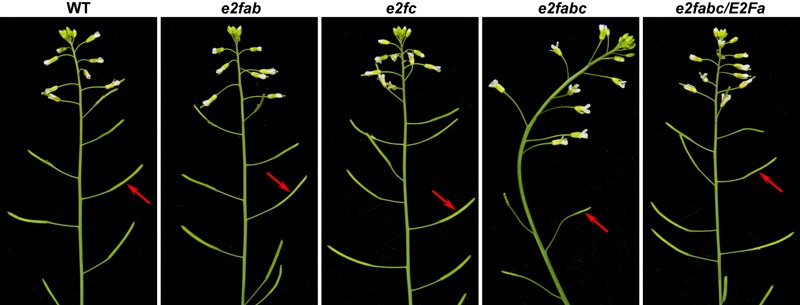
Arabidopsis E2Fs are crucial for plant fertility. Inflorescences of 5-week-old wild type (WT), *e2fab, e2fc, e2fabc*, and *E2Fa*-transgenic *e2fabc* (*e2fabc*/*E2Fa*) plants. Arrows indicate the silique.

Phylogenetic analysis showed that the sequences of three canonical Arabidopsis E2F proteins were similar to each other (**Figure 1C**). Consistently, our genetic analysis revealed that these canonical E2Fs functioned redundantly to activate plant effector-triggered cell death and immunity (Wang et al., 2014). In addition, they played a redundant role in plant fertility as the *e2fabc* triple mutant was sterile, whereas single and double *e2f* mutants were all fertile (**Figure 2** and Supplementary Figure S2) (Wang et al., 2014). The *e2f* mutant lines used for generating the *e2fabc* triple mutant are likely knockout lines as the insertion sites disrupt their DDs (Supplementary Figure S3). Although the reproductive development was severely compromised, the vegetative development of *e2fabc* mutant (germinated later than wild type plant) appeared normal (Supplementary Figure S4). We introduced the *E2Fa* gene, as well as *E2Fb* and *E2Fc*, into the *e2fabc* triple mutant and found that it fully restored the fertility of this mutant, confirming that mutations of the *E2F* genes are responsible for the sterility of *e2fabc* mutant (**Figure 2**, Supplementary Figure S5, and Supplementary Table S4).

### The Canonical E2Fs Are Essential for Pollen Mitosis During Male Gematogenesis

**FIGURE 3:**
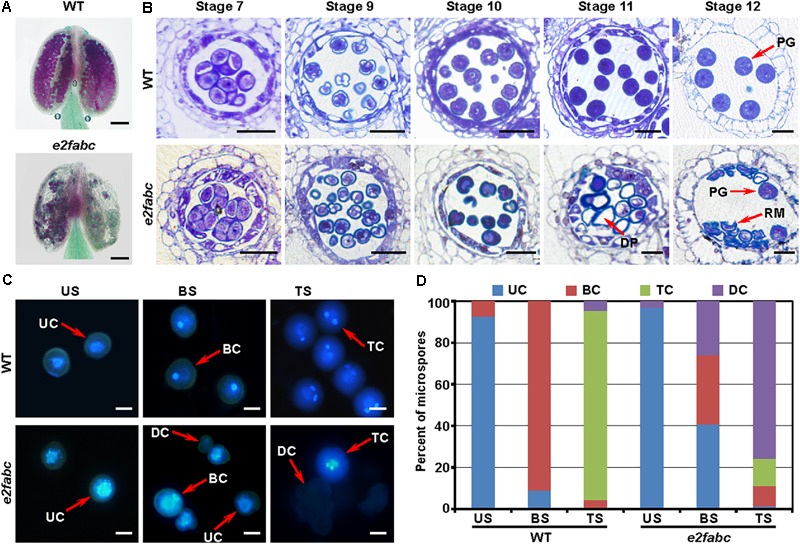
E2Fs are crucial for male gametogenesis. **(A)** The Alexander staining of wild type (WT) and *e2fabc* anthers. The aborted pollen are stained in green. Bar = 100 μm. **(B)** Semi-thin sections of WT and *e2fabc* anthers at the indicated stage are stained with toluidine blue. DP, degenerated pollen; PG, pollen grain; RM, remnant of microspore. Bar = 20 μm. **(C)** The DAPI staining of WT and *e2fabc* pollen at unicellular stage (US), bicellular stage (BS) and tricellular stage (TS). BC, bicellular cell; DC, degenerated cell; TC, tricellular cell; UC, unicellular cell. Bar = 5 μm. **(D)** Quantitative result of **(C)**. Experiments were conducted three times with similar results (*n* = 600∼800).

To explore the role of the canonical E2Fs in gametophytic control, we first examined the male gametogenesis which occurs in the anther. The reporters of *pE2F:E2F-VENUSs* showed that *E2Fs* were expressed in microspores, with the peak at the bicellular stage (Supplementary Figure S6). This expression pattern suggests that E2Fs play a role in male gametogenesis. The Alexander staining showed that in an *e2fabc* anther, some of pollen were viable, whereas the majority (81%, *n* = 600) of pollen were aborted (**Figure 3A**). The Arabidopsis anther development is divided into 14 stages (Sanders et al., 1999). The *e2fabc* mutant anther was not distinguishable from wild type anther until the stage 10, when the degeneration of tapetum initiated. The majority of the *e2fabc* microspores underwent cell death at the stage 11, when pollen mitosis I (PMI) initiated (**Figure 3B**). Consistently, the unicellular microspores were uniformly formed in both wild type and *e2fabc* anthers. The degeneration of *e2fabc* microspore started from the bicellular stage. At the tricellular stage, 75.6% of microspores were degenerated, whereas 13.4% of microspores developed into tricellular microspores in the *e2fabc* mutant (**Figures 3C,D**). These data suggest that the canonical E2Fs are required for the microspore development during the PMI progression.

### The Canonical E2Fs Are Essential for the Transition From Archesporial Cell to Megaspore Mother Cell During Female Sporogenesis

**FIGURE 4:**
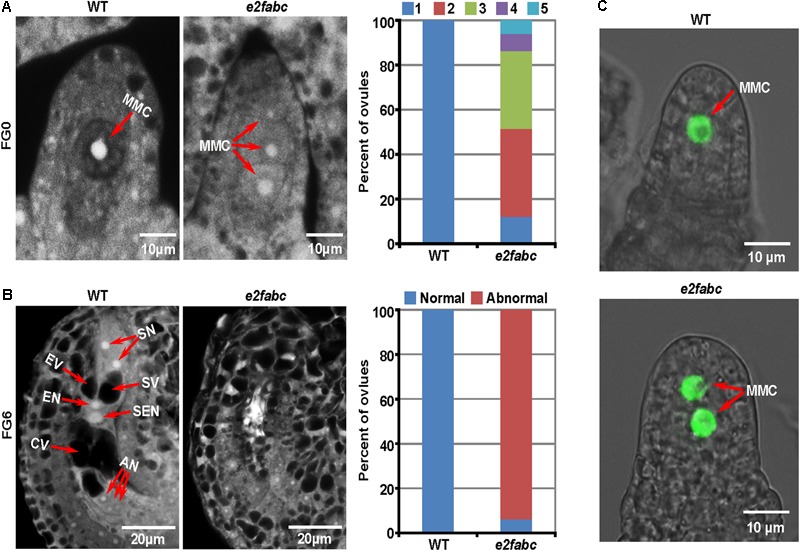
E2Fs are crucial for female gametogenesis. **(A)** Ovules of wild type (WT) and *e2fabc* plants at the FG0 (female gametophyte 0) stage. Ovules are classified by the number of MMC per ovule (from 1 to 5). The percent of ovules in different types is shown in the right panel. Experiments were performed three times with similar results (*n* = 60∼80). MMC, megaspore mother cell. **(B)** Ovules of WT and *e2fabc* plants at the FG6 stage. There are two types of ovules: normal (wild type) and abnormal (defective). The percent of ovules in different types is shown in the right panel. Experiments were performed three times with similar results (*n* = 60∼80). AN, antipodal nuclei; CV, central cell vacuole; EN, egg cell nucleus; EV, egg vacuole; SEN, secondary endosperm nucleus; SN, synergid nucleus; SV, synergid vacuole. **(C)** The MMC identity in WT and *e2fabc* ovules at the FG0 stage is shown by the marker *pKNU:KNU-VENUS*.

The female sporogenesis takes place in ovule. The AC is derived from a sub-epidermal somatic cell at the distal end of the ovule primordium. Usually, a single AC is selected to develop into a large MMC at the female gametophyte 0 (FG0) stage. Compared to the surrounding sporophytic cells, the MMC has a denser cytoplasm and a larger nucleus (Drews and Koltunow, 2011). Intriguingly, multiple MMCs (up to 5) were formed in an *e2fabc* mutant ovule. The majority of *e2fabc* mutant ovules produced 2-3 MMCs (**Figure 4A**). The subsequent female gametogenesis of *e2fabc* mutant was aborted (93.8%, *n* = 97) and the vacuole was severely impaired in the embryo sac (**Figure 4B** and Supplementary Figure S7). The cell identities were confirmed by a MMC marker *pKNU:KNU-VENUS* (**Figure 4C**) (Sun et al., 2014). The reporters of *pE2F:E2F-VENUSs* showed that the canonical E2Fs were expressed all over the ovule in the early development stages (before FG4 stage) (Supplementary Figure S8). Although about 5% of wild type ovules have two MMCs, it has never been observed that two female gametophytes are formed in one ovule in Arabidopsis, suggesting that the survival of one functional MMC per ovule is a strict rule required for the subsequent female gametophyte development (Drews and Koltunow, 2011). Occasionally, we observed a normal seven-celled female gametophyte formed in the *e2fabc* mutants (6.2% were normal at the FG7 stage, *n* = 97) (Supplementary Figure S7), which appears that the development of *e2fabc* ovule is delayed. This result is consistent with that the *e2fabc* mutant, especially during the late flowering stage, can set some seeds (about 20 seeds per plant, *n* = 50) (Supplementary Figure S9). These data suggest that the canonical E2Fs are required for the AC-MMC transition during female sporogenesis.

### The Canonical E2Fs Are Required for Gametophytic Control of Plant Gametogenesis and Suppression of Cell Cycle-Related Gene Expression

The dby both gametophytic and sporophytic genes. To investigate the role of the canonical E2Fs in plant gametophyte development, we analyzed the genetic transmission via gametophyte through reciprocal crosses between wild type and *e2fa*^+/-^ *e2fb*^-/-^ *e2fc*^-/-^ plants. The expected transmission efficiency for the normal gametes is 100%. As shown in **Table 1**, the transmission efficiency of *e2fa e2fb e2fc* triple mutant allele was 20.5 and 23.6% via male and female gametophytes, respectively, both of which were dramatically reduced as compared to the expected value, suggesting that the canonical E2Fs play a critical role in both male and female gametogenesis. Consistently, we observed that the majority of *e2fa*^+/-^ *e2fb*^-/-^ *e2fc*^-/-^ plants produced abnormal pollen and ovules (Supplementary Table S5). The deficiency of gametophyte development varied dramatically as the ratio of abnormal ovules was from 0 to 90%. The MMC marker *pKNU:KNU-VENUS* showed that 20∼75% of ovules contained multiple MMCs in *e2fa*^+/-^ *e2fb*^-/-^ *e2fc*^-/-^ plants. We further checked the *e2fb e2fc* double mutants and found that their gametophyte development was deficient to some extent, which was consistent with that more than 50% female gametophytes were defective in an *e2fa*^+/-^ *e2fb*^-/-^ *e2fc*^-/-^ plant (Supplementary Table S6). Similarly, genetic analysis of tetraploid plants (*rbr1*/*rbr1*/*rbr1*/*RBR1*, triplex for *rbr1*) found that regulation of the sporophytic development by RBR1 depended on the copy number of *RBR1* (Johnston et al., 2010). These data indicate that the gametogenesis is controlled by the canonical E2Fs from both male and female gametophyte in a dosage-dependent manner.

**Table 1:** Test of transmission efficiency through reciprocal crosses between wild type and *e2fa*^+/-^*e2fb*^-/-^*e2fc*^-/-^ plants.

Parents (♀ × ♂)^a^	Progeny		Total	TE^c^	*p*-value^d^
	*e2fa^+/-^e2fb^+/-^e2fc^+/-^*	*e2fa^+/+^e2fb^+/-^e2fc^+/-^*			
WT^b^ ×*e2fa*^+/-^ *e2fb*^-/-^ *e2fc*^-/-^	26	127	153	20.5%	<0.01
*e2fa*^+/-^ *e2fb*^-/-^ *e2fc*^-/-^× WT	29	123	152	23.6%	<0.01

*^*a*^♀×♂, female × male. ^**b**^WT, wild type plant. ^*c*^TE, transmission efficiency. TE = number of *e2fa^+/-^e2fb^+/-^e2fc^+/-^* progenies/number of *e2fa^+/+^e2fb^+/-^e2fc^+/-^* progenies × 100%. The expected TE for the normal gametes is 100%. ^*d*^The *p*-value is calculated by the χ^*2*^ test based on the expected TE of a 1:1 segregation ratio. χ^*2*^= Σ(observed value-expected value)^*2*^/expected value.*

E2Fs function as transcription factors to activates the expression of cell cycle regulators. To understand how E2Fs regulate plant germline development, we examine the expression of cell cycle-related genes. Genome-wide transcriptional profiling analysis revealed that a few of cell cycle regulators, including *RBR1, ORIGIN OF REPLICATION COMPLEX 1B* (*ORC1B*), *MINICHROMOSOME MAINTENANCE 8* (*MCM8*), *CYCLIN-DEPENDENT KINASE B1;1* (*CDKB1;1*) and *CELL DIVISION CONTROL 6* (*CDC6*), were upregulated by co-overexpression of *E2Fa* with *DPa* or down-regulated by a dominant-negative truncated *DP* gene (Ramirez-Parra et al., 2003; Vandepoele et al., 2005; Naouar et al., 2009). As shown in Supplementary Table S7, qPCR analysis was carried out to demonstrate the influence of *e2fabc* mutant on the expression of these genes in anthers (stage 10 ∼ stage 12) and ovules (FG0 ∼FG7). *TUBULIN BETA CHAIN 2* (*TUB2*) was used as an internal control. In addition, we first examined two other internal controls, *TUBULIN BETA 8* (*TUB8*) and *UBIQUITIN-CONJUGATING ENZYME 21* (*UBC21*), and validated that the expression of these internal controls was not altered by mutations of these *E2F* genes in anthers and ovules. Consistent to the phenotype, the expression of both male gametophyte-specific *MICROSPORE-SPECIFIC PROMOTER 2* (*MSP2*) and female gametophyte-specific *DOWNREGULATED IN DIF1 33* (*DD33*) was significantly downregulated in anthers and ovules, respectively, as both genes were about 10-fold reduced in *e2fabc* mutant (Honys et al., 2006; Steffen et al., 2007). Surprisingly, our results showed that the expression of all of the five E2F-target genes was upregulated in both anthers and ovules of *e2fabc* mutant, suggesting that the canonical Arabidopsis E2Fs play a negative role in transcription of these genes.

## Discussion

In mammals, the G1-S phase transition is controlled by the CDK-RB-E2F core cell cycle signaling pathway (Polager and Ginsberg, 2009). In the Arabidopsis genome, there are at least 50 cyclins, 12 CDKs, 18 CKIs and 8 E2Fs (Vandepoele et al., 2002; Wang G. et al., 2004). In contrast, the Arabidopsis genome only bears a single copy gene of *CDKA1* and *RB*. All types of these cell cycle regulators have been implicated in plant gametogenesis (Twell, 2011; Zhao et al., 2012). During cell cycle progression, CDK and E2F function as positive regulators, whereas RB acts as a negative regulator. The phenotype of *e2fabc* mutant appears earlier than that of *cdka1* and *rbr1* mutant (**Figures 3, 4**) (Ebel et al., 2004; Nowack et al., 2006). The redundancy between multiple genes (cyclins, CKIs, CDKs and E2Fs) and the lethality of a single copy gene (RB and CDKA1) have long hampered the genetic study on their functions. Fortunately, the *e2fabc* mutant is severely but not completely sterile, especially during the late flowering stage. A little bit of fertility and the normal vegetative development in the *e2fabc* triple mutant provide us with an opportunity to have in-depth study of the CDK-RB-E2F signaling pathway in plant germline development. Plant E2Fs control cell cycle as their mammalian counterpart (Vandepoele et al., 2005; Sozzani et al., 2006; Cheng et al., 2013; Liu et al., 2016). The canonical E2Fs had been found to play a distinct role during cell cycle progression based on the ectopic studies. Co-overexpression of E2Fa with DPa leads to activation of both mitosis and endoreduplication, whereas E2Fb and E2Fc act antagonistically as both co-overexpression of E2Fb with DPa and down-regulation of E2Fc by RNA interference induced mitosis but reduced the endoreduplication (De Veylder et al., 2002; Magyar et al., 2005; del Pozo et al., 2006). In addition, E2Fb is antagonistic to E2Fc in the transcription of the *DEL1* gene (Berckmans et al., 2011). Our data demonstrated that the three Arabidopsis canonical E2Fs played a redundant role in plant fertility (**Figures 1–3**), in addition to plant effector-triggered cell death and immunity (Wang et al., 2014).

E2F acts as a transcription factor which is an executor of the CDK-RB-E2F signaling pathway on the expression of target genes. Our data showed that the canonical E2Fs were required for plant germline development, especially the pollen mitosis and the AC-MMC transition (**Figures 3, 4**). The underlying mechanism of how PMI is regulated remains unknown. It seems that the cytokinesis plays a crucial role in the asymmetrical cell division of PMI as most of the identified mutants with PMI defects are related to microtubule (Twell et al., 2002; Pastuglia et al., 2006). Plant RB, E2F and MYB3R are proposed to be the member of a DREAM complex which plays a critical role in maintaining cell quiescence (Magyar et al., 2016). Arabidopsis MYB3Rs were found to regulate cytokinesis through activation of the *KNOLLE* transcription (Haga et al., 2007), suggesting that E2F could be involved in cytokinesis through its partner MYB3R. In the meantime, the underlying mechanism of how the MMC develop also remains largely unknown. MAC1(MULTIPLE ARCHESPORIAL CELLS 1) encodes a leucine-rich repeat containing receptor-like kinase (LRR-RLK). MSP1 (MULTIPLE SPOROCYTE 1) encodes a putative ligand of MAC1. MAC1 and MSP1 control the transition from somatic to germline fate as mutation of maize MAC1 and rice MSP1 resulted in multiple ACs (Sheridan et al., 1996; Nonomura et al., 2003). Previously, a combined analysis of laser-assisted microdissection and microarray revealed that MNEME (MEM) was preferentially expressed in the MMC. MEM encodes an ATP-dependent RNA helicase. Mutation of MEM leads to the multiple MMCs. Like MMCs of the *e2fabc* mutant, those of the *mem* mutant are also aborted after the FG0 stage. The male gametophyte development of *mem* mutant is not as dramatically affected as that of *e2fabc* mutant. Intriguingly, the microarray data of microdissected cells show that all of the canonical E2Fs are preferentially expressed in MMCs as compared to ovules (Supplementary Figure S10) (Schmidt et al., 2011), which is in good agreement with our data that the canonical E2Fs play a crucial role in the MMC initiation. The stem cell regulator WUSCHEL (WUS) plays a role in the AC-MMC transition. RBR1, the repressor of E2Fs, was found to control the AC-MMC transition through repression of the WUS expression (Lieber et al., 2011; Zhao et al., 2017). It has also been shown that the MMC initiation is controlled by a clay of Argonaute (AGO) genes including AGO4, AGO6, AGO8 and AGO9 in Arabidopsis. Mutations of these genes give rise to multiple MMCs per ovule. Among the multiple MMCs, only one MMC is functional and further develops into a gametophyte (Olmedo-Monfil et al., 2010; Hernandez-Lagana et al., 2016). In contrast to the *e2fabc* mutants, these *ago* mutants are fertile, suggesting that the canonical E2Fs are required for not only the AC-MMC transition at the FG0 stage but also the gametophyte development after the FG0 stage.

AGO protein is an RNA Slicer that functions in epigenetic regulation through the RNA-dependent DNA methylation (RdDM) signaling pathway. It interacts with transcripts produced by Polymerase V (Pol V) to recruit *de novo* DNA methyltransferases, such as DOMAINS REARRANGED METHYLTRANSFERASE 2 (DRM2) histone methyltransferases and chromatin remodelers, to silence a gene (Law and Jacobsen, 2010). It has been observed that the AC-MMC transition in Arabidopsis was accompanied by a large-scale chromatin reprogramming (She et al., 2013), suggesting that control of the AC-MMC transition by AGOs may be attributed to the epigenetic regulation. E2F forms a complex with RB which represses the E2F activity through either physical interaction (masking activation domain) or epigenetic regulation. RB recruits chromatin-remodeling factors and chromatin modifiers such as the SWITCH/SUCROSE NON-FERMENTABLE (SWI-SNF) complex, HISTONE DEACETYLASES (HDACs), and SET-DOMAIN-CONTAINING HISTONE METHYLTRANSFERASES (HMTases) to epigenetically repress the E2F-target genes (Robertson et al., 2000; Zhang et al., 2000). Our data showed that the repression of E2F-target genes, which may be controlled by the RB-E2F complex, was released in *e2fabc* mutant (Supplementary Table S7). This is consistent with that mutations of both E2Fs and its repressor RBR1 in Arabidopsis lead to multiple MMCs (**Figure 4**; Zhao et al., 2017). These evidences strongly support that epigenetic regulation plays a critical role in the AC-MMC transition. The variation of female gametophyte deficiency in the progeny of an *e2fa*^+/-^ *e2fb*^-/-^ *e2fc*^-/-^ plant may result from a combined effect of quantitative (copy of E2F) and epigenetic factors.

## Author Contributions

XY, SW, and ZY conceived and designed the research project. XY, HY, YZ, SW, JX, TW, and TS performed the experiments. SW and XY wrote the manuscript.

## Conflict of Interest Statement

The authors declare that the research was conducted in the absence of any commercial or financial relationships that could be construed as a potential conflict of interest.

## Footnotes

1 http://www.cambia.org
