# Supplementary material for: The Canonical E2Fs Are Required for Germline Development in Arabidopsis
Source: Front Plant Sci. 2018 May 15;9:638. doi: 10.3389/fpls.2018.00638 (PMC5962754; doi:10.3389/fpls.2018.00638)
Supplement: Supplementary file 1 [file Image_1.pdf]

SUPPLEMENTARY MATERIAL

# The Canonical E2Fs Are Required for Germline Development in Arabidopsis

**Xiaozhen Yao[1], Huidan Yang[1], Yingxiu Zhu, Jingshi Xue, Tianhua Wang, Teng Song, Zhongnan Yang, Shui Wang***

***Correspondence author:** Shui Wang: shuiwang@shnu.edu.cn

## Supplementary Figures and Tables

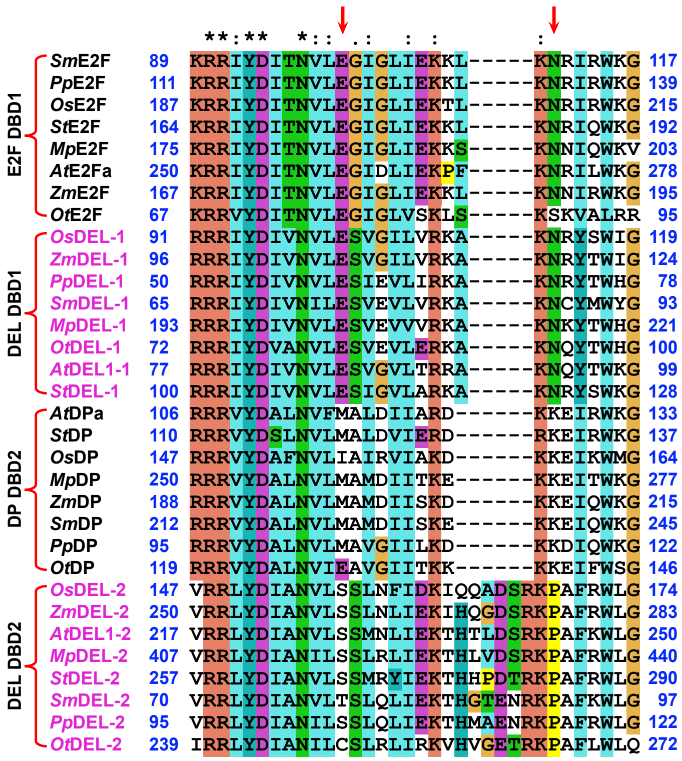


**Supplementary Figure S1.** Alignment of DBD of plant E2F proteins using ClustalX2 (http://www.clustal.org/). E2F family proteins are classified into three categories: E2F, DEL and DP. DBD, DNA-binding domain. E2Fs possess one DBD (E2F DBD1), DELs possess two DBDs (DEL DBD1 and DEL DBD2), and DPs possess one DBD (DP DBD2). Arrows indicate that two amino acids, glutamate (E) and asparagine (N), in DBD1 are shared between E2F and DEL proteins. The GenBank accession numbers of E2Fs analyzed in this figure are as listed in Supplementary Table 3. *At*, *Arabidopsis thaliana*; *Mp*, *Micromonas pusilla*; *Os*, *Oryza sativa Japonica*; *Ot*, *Ostreococcus tauri*; *Pp*, *Physcomitrella patens*; *Sm*, *Selaginella moellendorffii*; *St*, *Solanum tuberosum*; *Zm*, *Zea mays*.

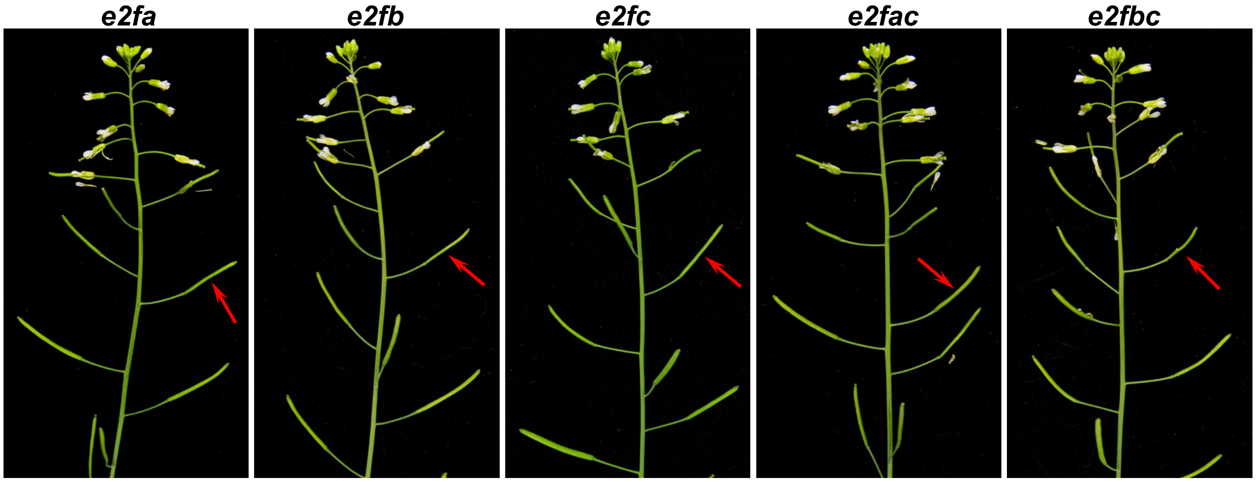


**Supplementary Figure S2** | The single and double *e2f* mutants are fertile. Inflorescences of 5-week-old *e2fa*, *e2fb*, *e2fc*, *e2fac*, and *e2fbc* mutants. The images were photographed at the same time as Figure 2.

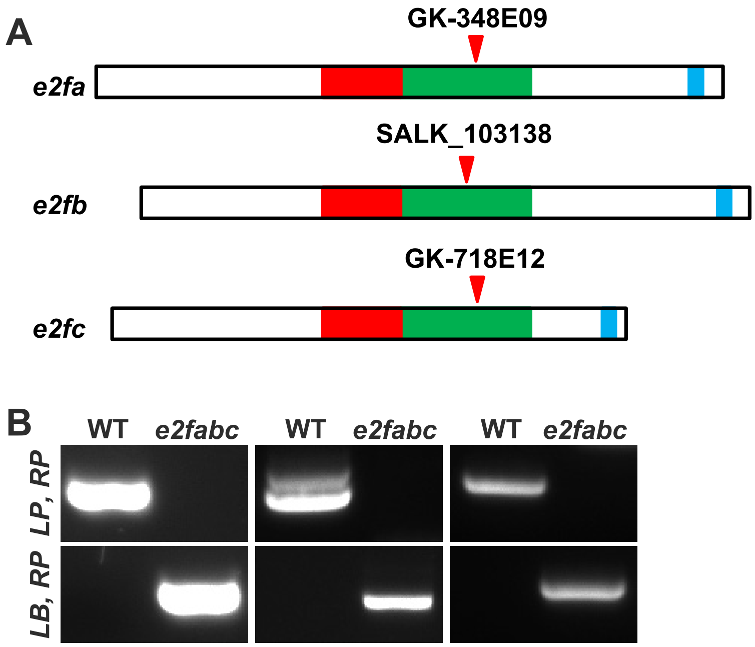


**Supplementary Figure S3. The position and confirmation of T-DNA in the *e2f* mutants. (A)** The T-DNA insertion lines of *e2fa* (GK-348E09), *e2fb* (SALK_103138), and *e2fc* (GK-718E12) mutants. Arrow heads indicate the T-DNA insertion sites. DNA-binding domain (DBD), dimerization domain (DD) and RB-binding domain are represented in red, green and blue box, respectively. (B) Genotyping of wild type and *e2fabc* plants. LP and RP are the gene-specific forward and reverse primers, respectively. LB is a T-DNA forward primer. The primers used for genotyping are listed in Supplementary Table 1.

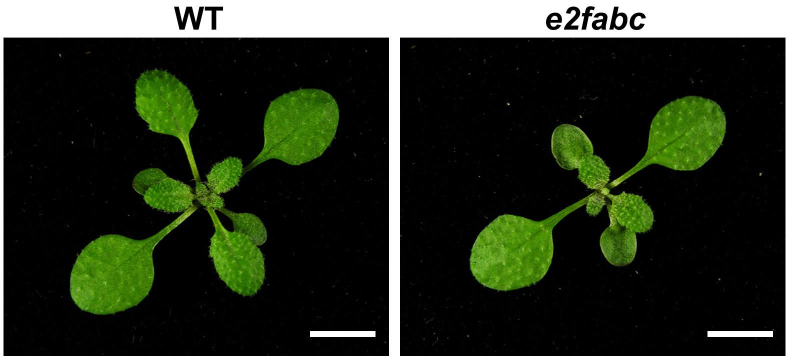


**Supplementary Figure S4.** The *e2fabc* mutant exhibits a normal vegetative development. Two-week-old wild type and *e2fabc* plants. Bar = 1 cm.

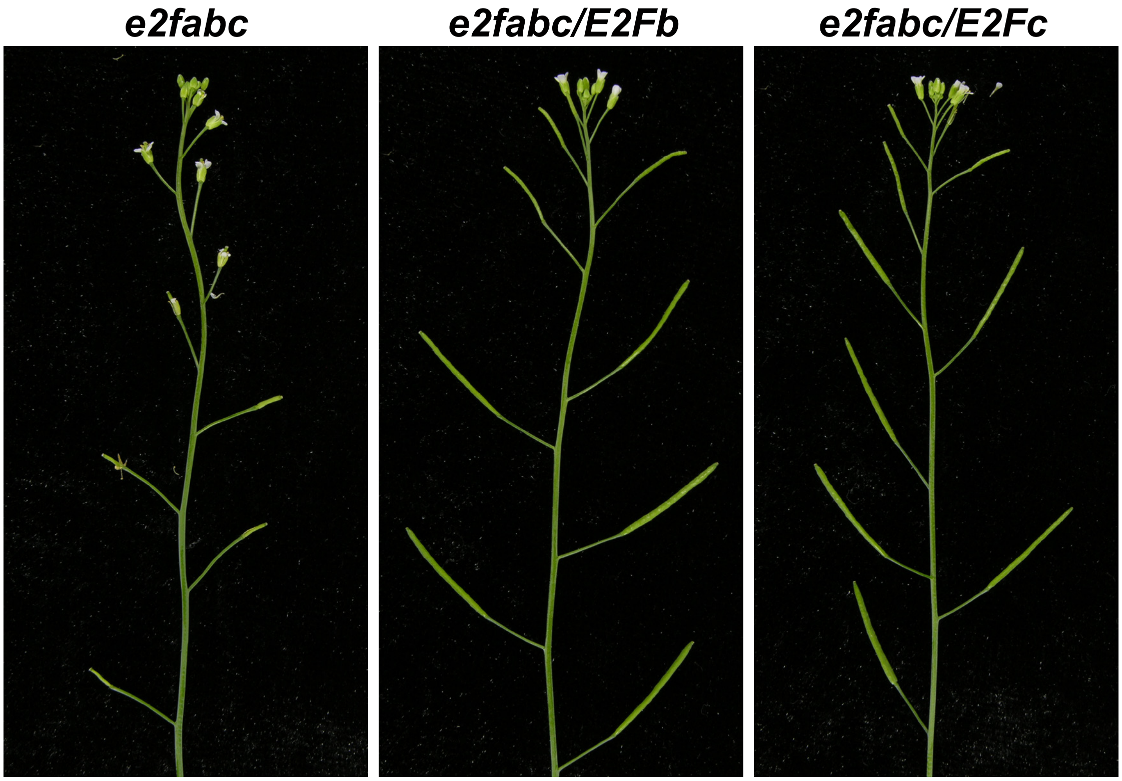


**Supplementary Figure S5.** The sterility of *e2fabc* mutant is rescued by the genes of *E2Fb* (*e2fabc*/*E2Fb*) or *E2Fc* (*e2fabc*/*E2Fc*).

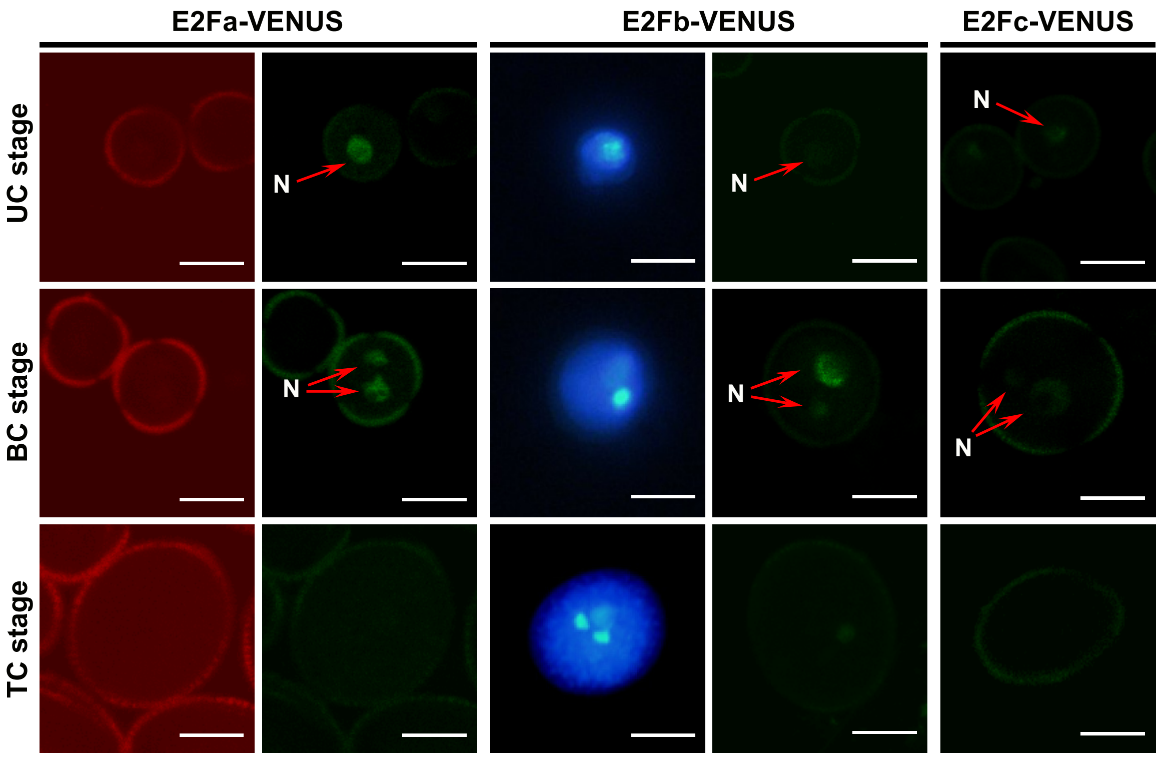


**Supplementary Figure S6.** The expression of the canonical E2Fs in pollen is shown by the reporter of *E2F* promoter-driven *E2F* and *VENUS* fusion genes (*pE2F:E2F-VENUS*). The fluorescence of VENUS was excited at 448 nm. For E2Fa-VENUS, the fluorescence was excited at 545 nm to show the autofluorescence of pollen wall (on the left). For E2Fb-VENUS, the staining with DAPI was carried out to show the nucleus at the same developmental stage (on the left). BC, bicellular cell; N, nucleus; TC, tricellular cell; UC, unicellular cell. Bar = 10 μm.

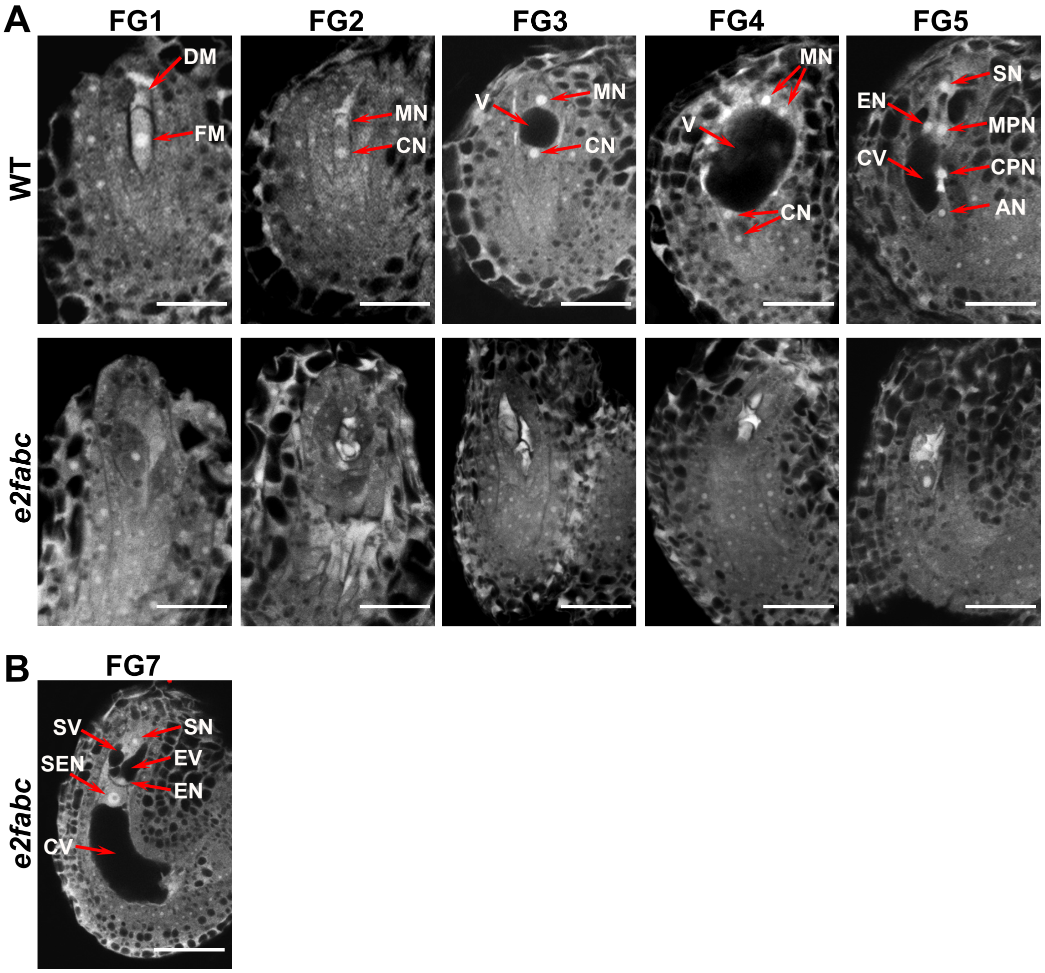


**Supplementary Figure S7.** The phenotype of ovule development. **(A)** Ovules of wild type and *e2fabc* plants at the FG1 through FG5 stages. The female gametogenesis of *e2fabc* mutant is disrupted since the FG0 stage. (B) A normal ovule of *e2fabc* mutant. AN, antipodal nucleus; CN, chalazal nucleus; CV, central cell vacuole; CPN, chalazal polar nucleus; DM, degenerating megaspore; EN, egg nucleus; EV, egg vacuole; FM, functional megaspore; MN, micropylar nucleus; MPN, micropylar polar nucleus; SN, synergid nucleus; SV, synergid vacuole; V, vacuole. Bar = 20 μm.

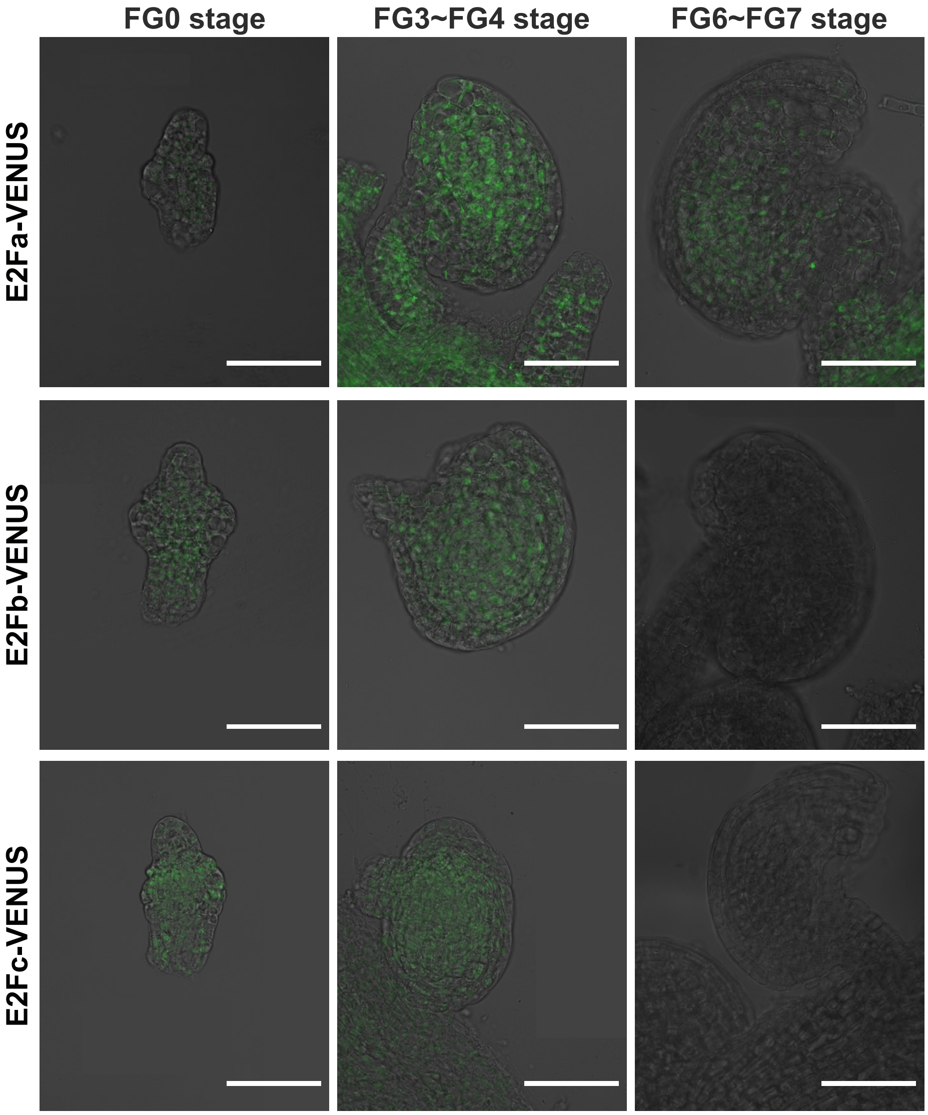


**Supplementary Figure S8.** The expression of the canonical E2Fs in ovule is shown by the the reporter of *E2F* promoter-driven *E2F* and *VENUS* fusion genes (*pE2F:E2F-VENUS*). Bar = 50 μm.

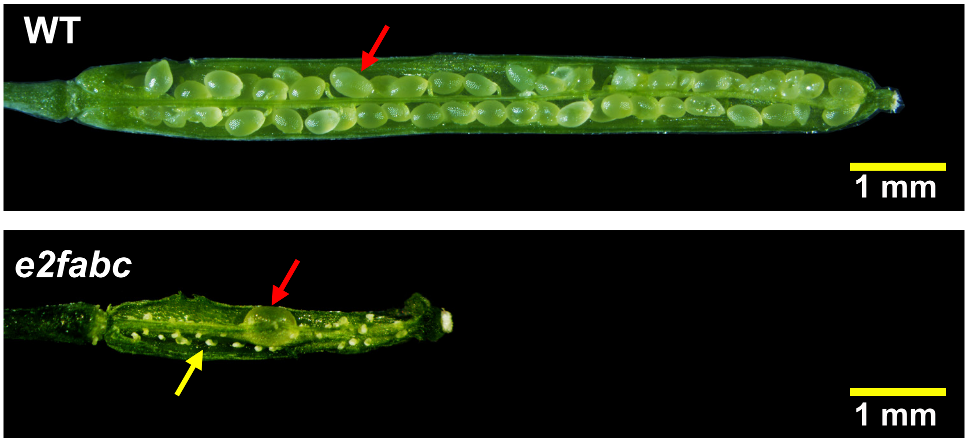


**Supplementary Figure S9 |** The *e2fabc* triple mutant is sterile. An opened silique at 7 days after pollination (DAP) in wild type and *e2fabc* plant (n=30) is shown. The red arrow indicates the normal seed while the yellow arrow indicates the unfertilized ovule.

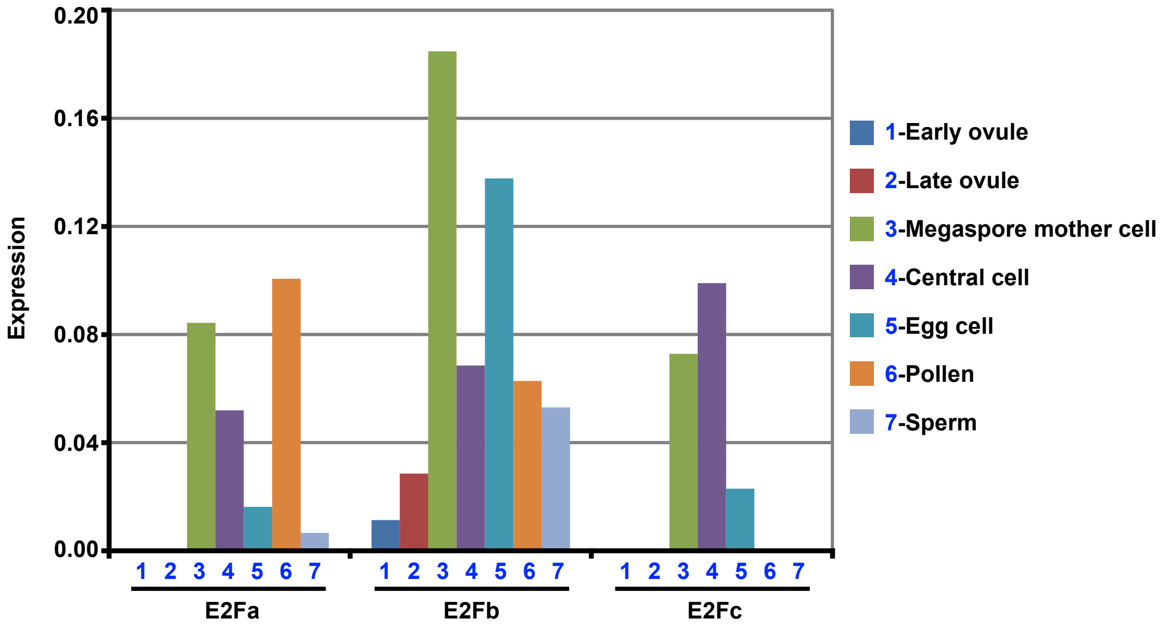


**Supplementary Figure S10.** *E2F*s are preferentially expressed in MMCs. The expression of *E2Fa*, *E2Fb* and *E2Fc* in early ovule, late ovule, megaspore mother cell, central cell, egg cell, pollen and sperm. These samples were isolated through the laser-assisted microdissection and the expression was analyzed by microarray. These data are extracted from a published paper (Schmidt et al., 2011).

**Supplementary Table S1** Primers used for constructions and quantitative PCR

| **Name** | **Sequence (5'-3')** |
|---|---|
| GABI-o8409-LB | ATATTGACCATCATACTCATTGC |
| LBb1-3 | ATTTTGCCGATTTCGGAAC |
| e2fa-GABI-LP | TCCTGGTGGTGGAGTACTCAC |
| e2fa-GABI-RP | GGTTTCCAGGTCTGTCTTTCC |
| e2fb-SALK-LP | TGCGAACTCTGTTATGCAATG |
| e2fb-SALK-RP | TTCATCAGCCTGAGGAATGTC |
| e2fc-GABI-LP | TCTATCGTGAACAAAAACAGATGCT |
| e2fc-GABI-RP | CTGAGGAAAGCAGGTTGGATGATCT |
| E2Fa-1300-SalI-F | ggtacccggggatcctctagaCATGAAGCTTGTGCTGCGAA |
| E2Fa-1300-SalI-R | gccaagcttgcatgcctgcagCAATTCCGGGATCACCCCTCA |
| E2Fb-1300-SalI-F | ggtacccggggatcctctagaGATGACCCTGATCTTGCCCA |
| E2Fb-1300-SalI-R | gccaagcttgcatgcctgcagGAGATTCAGCATTGCTCATTG |
| E2Fc-1300-SalI-F | ggtacccggggatcctctagaGTCACATGCCATAGGGACGA |
| E2Fc-1300-SalI-R | gccaagcttgcatgcctgcagCGAATCTGCTCTATACCTTCTCT |
| VENUS-SalI-F | tatgtcgacATGGTGAGCAAGGGCGAGGAGCTGT |
| VENUS-PstI-R | tatctgcagTTACTTGTACAGCTCGTCCATGC |
| E2Fa-VENUS-F | ggtacccggggatcctctagaCATGAAGCTTGTGCTGCGAA |
| E2Fa-VENUS-R | agctcctcgcccttgctcaccatTCTCGGGGTTGAGTCAACAG |
| E2Fb-VENUS-F | ggtacccggggatcctctagaGATGACCCTGATCTTGCCCA |
| E2Fb-VENUS-R | agctcctcgcccttgctcaccatGCTACCTGTAGGTGATCTCG |
| E2Fc-VENUS-F | ggtacccggggatcctctagaGTCACATGCCATAGGGACGA |
| E2Fc-VENUS-R | agctcctcgcccttgctcaccatGCTGTTGAAGTTGCTCCATAAATC |

**Supplementary Table S1** Primers used for constructions and quantitative PCR (continued)

| Name | Sequence (5'-3') |
|---|---|
| CDC6-RT-F | GCACCGTCAACTGTTGTTTG |
| CDC6-RT-R | CCTGCTTTGCCCATTCTTCA |
| CDKB1;1-RT-F | CTGCGTGACTGGCATGTTTA |
| CDKB1;1-RT-R | CGGCTGGATTGTACTTGAGC |
| RBR1-RT-F | GGTCTGTCAGTGTCTGGTGA |
| RBR1-RT-R | AGTCTGTTCTCCCAGTCTGC |
| MCM8-RT-F | GCGTTGCTAGAAGCAATGGA |
| MCM8-RT-R | AGAGAAGGGCAGCACTCATT |
| ORC1-RT-F | GGCTGAATTGGCTGATGGAG |
| ORC1-RT-R | GAGAGTTCGCACCTCCTCTT |
| DD33-RT-F | TGATCCGATGTGTCCTGGAG |
| DD33-RT-R | CAAGCAGTTGGTGGAACATCA |
| MSP2-RT-F | ACGGCGATTAGAGAAGCGTA |
| MSP2-RT-R | ACTGCTCGATTCCAACTCCA |
| TUB2-RT-F | ATCGATTCCGTTCTCGATGT |
| TUB2-RT-R | ATCCAGTTCCTCCTCCCAAC |
| TUB8-RT-F | GATTTCAAAGATTAGGGAAGAGTA |
| TUB8-RT-R | GTTCTGAAGCAAATGTCATAGAG |
| UBC21-RT-F | TGGACCGCTCTTATCAAAGGA |
| UBC21-RT-R | CAAGCAGGACTCCAAGCATT |

**Supplementary Table S2** The members of E2F family in plants[a]

| **Species** | **Group** | **E2F** | **DEL** | **DP** | **Total** |
|---|---|---|---|---|---|
| *Arabidopsis* | Eudicot | 3 | 3 | 2 | 8 |
| *Solanum tuberosum* | Eudicot | 4 | 1 | 2 | 7 |
| *Oryza sativa Japonica* | Monocot | 4 | 2 | 3 | 9 |
| *Zea mays* | Monocot | 5 | 3 | 3 | 11 |
| *Selaginella moellendorffii* | Lycopodiophyta | 4 | 2 | 1 | 7 |
| *Physcomitrella patens* | Bryophyta | 4 | 3 | 3 | 10 |
| *Micromonas pusilla* | Algae | 1 | 1 | 1 | 3 |
| *Ostreococcus tauri* | Algae | 1 | 1 | 1 | 3 |

[a]Plant E2F family proteins are classified into three categories: E2F, DEL and DP.

**Supplementary Table S3** The GenBank accession numbers of E2F family proteins in plants

| **Species** | **Member** | **Accession number** |
|---|---|---|
| *Solanum tuberosum* | St-1 (StDP) | XP_006341038 |
| | St-2 | XP_006362266 |
| | St-3 (StE2F) | XP_006362858 |
| | St-4 | XP_006361100 |
| | St-5 | XP_006348486 |
| | St-6 | XP_006342039 |
| | St-7 | XP_006338510 |
| | St-8 (StDEL) | XP_006345001 |
| *Oryza sativa Japonica* | Os-1 (OsDP) | XP_015631684 |
| | Os-2 (OsE2F) | XP_015623378 |
| | Os-3 | BAD27913 |
| | Os-4 (OsDEL) | XP_015626334 |
| | Os-5 | XP_015628001 |
| | Os-6 | BAH92463 |
| | Os-7 | XP_015637107 |
| | Os-8 | XP_015643420 |
| | Os-9 | EEE52804 |
| | Os-10 | ABA96516 |

**Supplementary Table S3** The GenBank accession numbers of E2F family proteins in plants (continued)

| Species | Member | Accession number |
|---|---|---|
| *Zea mays* | Zm-1 (ZmE2F) | NP_001130952 |
| | Zm-2 | AQK71576 |
| | Zm-3 | NP_001146160 |
| | Zm-4 | ACN30613 |
| | Zm-5 (ZmDEL) | NP_001307734 |
| | Zm-6 | AQK74714 |
| | Zm-7 | XP_008649275 |
| | Zm-8 (ZmDP) | NP_001169170 |
| | Zm-9 | XP_008677672 |
| | Zm-10 | NP_001347178 |
| *Selaginella moellendorffii* | Sm-1 (SmDEL) | XP_002981896 |
| | Sm-2 | XP_002986017 |
| | Sm-3 | XP_002989083 |
| | Sm-4 | XP_002983689017 |
| | Sm-5 (SmE2F) | XP_002993789 |
| | Sm-6 | XP_002993271 |
| | Sm-7 (SmDP) | XP_002989568 |

**Supplementary Table S3** The GenBank accession numbers of E2F family proteins in plants (continued)

| Species | Member | Accession number |
|---|---|---|
| *Physcomitrella patens* | Pp-1 | BAK64058 |
| | Pp-2 (PpDP) | PNR42845 |
| | Pp-3 (PpE2F) | PNR61872 |
| | Pp-4 | XP_001774130 |
| | Pp-5 (PpDEL) | PNR31992 |
| | Pp-6 | PNR30651 |
| | Pp-7 | PNR60617 |
| | Pp-8 | PNR28424 |
| | Pp-9 | PNR57322 |
| | Pp-10 | PNR55271 |
| | Pp-11 | BAK64059 |
| *Micromonas pusilla* | Mp-1 (MpE2F) | XP_003056615 |
| | Mp-2 (MpDEL) | XP_003058647 |
| | Mp-3 | XP_003062533 |
| | Mp-4 (MpDP) | XP_003058096 |
| *Ostreococcus tauri* | Ot-1 (OtDEL) | XP_003080858 |
| | Ot-2 (OtDP) | AAV68607 |
| | Ot-3 (OtE2F) | AAV68605 |

**Supplementary Table S4** The deficiency of seed development in wild type, *e2f* and *E2F*-transgenic *e2f* plants

| **Genotype** | **Aborted seeds** |
|---|---|
| WT | 0% (n=163) |
| *e2fa* | 1.3% (n=155) |
| *e2fb* | 1.2% (n=166) |
| *e2fc* | 0% (n=148) |
| *e2fa e2fb* | 7.6% (n=262) |
| *e2fa e2fc* | 4.5% (n=267) |
| *e2fb e2fc* | 5.1% (n=253) |
| *e2fabc* | 99.4% (n=338) |
| *e2fabc/E2Fa* | 4.5% (n=156) |
| *e2fabc/E2Fb* | 4.0% (n=149) |
| *e2fabc/E2Fc* | 6.2% (n=162) |

**Supplementary Table S5** The deficiency of pollen and ovule development

| **Class** | **No. of plants** | **Aborted pollen** | **Abnormal ovules** |
|---|---|---|---|
| I | 4 | 46.64% (n=892) | 91.6% (n=70) |
| II | 7 | 20.28% (n=1085) | 29.6% (n=71) |
| III | 4 | 0.6% (n=805) | 0% (n=53) |

**Supplementary Table S6** The deficiency of female germline development in *e2f* mutants

| **F2 progeny**[a] | | **Number of MMCs**[b] | | | | **Abnormal ovules** |
|---|---|---|---|---|---|---|
| **Genotype** | **No.** | **1** | **2** | **3** | **Abnormal (%)** | |
| *e2fb e2fc* | 1 | 9 | 2 | | 18.2% | 12.8% (n=128) |
| | 2 | 9 | 1 | | 10% | 8.5% (n=164) |
| | 3 | 12 | | | 0% | 2.9% (n=136) |
| | 4 | No fluorescence[c] | | | | 6.7% (n=313) |
| *e2fa*$^{+/-}$ *e2fb*$^{-/-}$ *e2fc*$^{-/-}$ | 1 | 12 | 3 | | 20% | 45.4% (n=99) |
| | 2 | 6 | 2 | | 25% | 33.9% (n=121) |
| | 3 | 3 | 9 | | 75% | 88.9% (n=162) |
| | 4 | 3 | 9 | 1 | 76.9% | 71% (n=183) |
| | 5 | No fluorescence | | | | 65.3% (n=219) |
| | 6 | No fluorescence | | | | 86.5% (n-282) |
| *e2fa e2fb e2fc* | 1 | 2 | 7 | 2 | 81.8% | 100% (n=136) |
| | 2 | 2 | 8 | 1 | 81.8% | 98.75% (n=160) |
| | 3 | No fluorescence | | | | 98.3% (n=120) |

[a]F2 progeny of the cross between the *e2fa e2fb e2fc* triple mutant and the reporter of *KNU:VENUS*.

[b]The number of megaspore mother cells (MMCs) are indicated by the reporter of *KNU:VENUS*.

[c]Plant without the reporter of *KNU:VENUS*.

**Supplementary Table S7** The expression of cell cycle-related genes and germline cell-specific genes in wild type and *e2fabc* plants[a]

| **Gene** | **AGI** | **Fold change (*e2fabc*/WT)** [b] | |
|---|---|---|---|
| | | **Anthers**[c] | **Ovules**[d] |
| *TUB8* | AT5G23860 | 0.84±0.03 | 1.03±0.12 |
| *UBC21* | AT5G25760 | 1.21±0.07 | 0.92±0.01 |
| *DD33* | AT2G20070 | | 0.09±0.04 |
| *MSP2* | AT5G46795 | 0.08±0.01 | |
| *RBR1* | AT3G12280 | 2.83±0.06 | 3.51±1.08 |
| *ORC1B* | AT4G12620 | 4.12±1.56 | 1.66±0.20 |
| *MCM8* | AT3G09660 | 4.77±1.64 | 4.33±0.72 |
| *CDKB1;1* | At3g54180 | 5.27±1.15 | 1.96±0.56 |
| *CDC6* | AT2G29680 | 1.49±0.82 | 4.05±0.93 |

[a]*TUBULIN BETA CHAIN 2* (*TUB2*) was used as an internal control. Experiments were conducted in triplicates. *TUB8*, *TUBULIN BETA 8*; *UBC21*, *UBIQUITIN-CONJUGATING ENZYME 21*; *DD33*, *DOWNREGULATED IN DIF1 33*; *MSP2*, *MICROSPORE-SPECIFIC PROMOTER 2*; *RBR1*, *RETINOBLASTOMA-RELATED 1*; *ORC1B*, *ORIGIN OF REPLICATION COMPLEX 1B*; *MCM8*, *MINICHROMOSOME MAINTENANCE 8*; *CDKB1;1*, *CYCLIN-DEPENDENT KINASE B1;1*; *CDC6*, *CELL DIVISION CONTROL 6*.

[b]Fold change (*e2fabc*/WT) = mean ± standard error.

[c]The WT and *e2fabc* anthers from stage 10 to stage 12 were used for qPCR.

[d]The WT and *e2fabc* ovules from FG0 to FG7 were used for qPCR.
